# Diabetes and covid-19: more than the sum of two morbidities

**DOI:** 10.11606/s1518-8787.2020054002577

**Published:** 2020-05-22

**Authors:** Bianca de Almeida Pititto, Sandra Roberta G. Ferreira

**Affiliations:** I Universidade Federal de São Paulo Escola Paulista de Medicina Departamento de Medicina Preventiva São PauloSP Brasil Universidade Federal de São Paulo. Escola Paulista de Medicina. Departamento de Medicina Preventiva. São Paulo, SP, Brasil; II Universidade de São Paulo Faculdade de Saúde Pública São PauloSP Brasil Universidade de São Paulo. Faculdade de Saúde Pública. São Paulo, SP, Brasil

**Keywords:** Diabetes Mellitus, epidemiology, Coronavirus Infections, complications, Comorbidity, Developing Countries

## Abstract

The coronavirus disease 2019 (covid-19) pandemic has caused a public health emergency worldwide. Risk, severity and mortality of the disease have been associated with non-communicable chronic diseases, such as diabetes mellitus. Accumulated evidence has caused great concern in countries with high prevalence of this morbidity, such as Brazil. This text shows the picture of diabetes in Brazil, followed by epidemiological data and explanatory hypothesis for the association between diabetes and covid-19. We emphasized how the burden of these two morbidities in a middle-income country has aggravated this pandemic scenario. The comprehension of this association and biological plausibility may help face this pandemic and future challenges.

## INTRODUCTION

On March 11^th^, 2020, the World Health Organization declared the outbreak caused by the novel coronavirus (SARS-CoV-2), the coronavirus disease 2019 (covid-19), as pandemic. The disease is responsible for more than 180 thousand deaths worldwide, with approximately 4,000 occurring in Brazil until the end of April 2020 (https://coronavirus.saude.gov.br/). Fatality rates have varied among countries depending on factors that influence both numbers of confirmed cases and registered deaths, as well as the proportion of at-risk individuals in the population (older adults, people with chronic diseases), access to health services, availability of accurate diagnostic tests and resources to deal with severe and critical cases (ICU, mechanical ventilators, trained health care professionals).

A huge number of studies has helped clarify clinical profiles of SARS-CoV-2 infection, showing consistently that the presence of chronic morbidities such as diabetes mellitus (DM) and its associated diseases (obesity, hypertension, and cardiovascular disease) represent major risk factors for the severity and prognosis of the disease, in addition to advanced age. Accumulated evidence has caused a great concern in countries with high prevalence of these morbidities such as Brazil.

This text shows the picture of diabetes in Brazil, followed by epidemiological data and explanatory hypothesis for the association between diabetes and covid-19. We also emphasize how the burden of these two morbidities in a middle-income country may aggravate this pandemic scenario.

### The Actual Burden of Diabetes in Brazil

Brazil is a typical example of the alarming picture of DM in emergent societies for this century, being the fourth country with the largest number of diabetic people. In 2019, it was estimated that 16.8 million individuals, aged between 20 and 79 years had DM in Brazil, with projection of 55% increase until 2045^1^ .

In the 1980s, a multicenter prevalence study, including 21,847 Brazilians aged between 30 and 69 years submitted to oral glucose tolerance test, found that 7.6% had DM^2^ . Most individuals had type 2 DM as occurs worldwide. We also found that almost half of individuals with DM were unaware of their condition. Even more impressive was the rate of undiagnosed DM registered in 2014 in the Estudo Longitudinal de Saúde do Adulto (ELSA-Brasil—Brazilian Longitudinal Study of Adult Health) that found the same proportion in 15,000 participants of a non-representative sample^3^ .

A nationwide system of surveillance of risk and protective factors for chronic diseases by telephone survey (VIGITEL) in Brazil has yearly provided epidemiological data about non-communicable chronic diseases (NCCD) in individuals older than 18 years. A rate of 5.5% of self-reported DM was described in 2016 and two years later it reached 8.0%^4^ . Since previous studies have shown that half of diabetic Brazilians were undiagnosed, those frequencies were certainly underestimated. As expected, rates vary according to age group, but also according to schooling, being 23.1% for individuals older than 65 years and 15.2% for those with less than 8 years of total schooling^4^ .

Another aspect of DM in Brazil refers to its metabolic control. A nationwide cross-sectional study, evaluating 5,750 patients with type 2 DM attending the public health care system between 2006 and 2011^5^ , reported a mean glycated hemoglobin (HbA1c) of 8.6% (SD 2.2). Targets (HbA1c <7%) recommended by scientific societies, such as the Brazilian Society of Diabetes, are achieved by a minority of patients even when a less strict target is considered. Only 48.5% of Brazilian patients sustain HbA1c < 8% which is poorer glycemic control than the observed in Europe and the USA. A multicenter study conducted in private health care system of Latin American countries showed that, among 878 Brazilian patients, approximately 40% had HbA1c levels higher than 7%^6^ .

As in other countries, in Brazil, NCCD are responsible for practically two thirds of the causes of death, being 5.2% due to DM^7^ . Furthermore, DM represents an important risk factor for cardiovascular disease (CVD), which accounts for 31.3% of deaths in Brazil^8^ .

This scenario of high DM prevalence (mainly in older adults and low socioeconomic stratus) associated with late diagnosis and poor glycemic control has a deleterious effect on the occurrence of long-term complications of the disease – macroangiopathy, retinopathy, nephropathy and neuropathy. Considering the covid-19 pandemic, global concerns on public health systems visibly increased, worsening the prognosis of diabetic individuals affected by the SARS-Cov-2 infection.

### Diabetes and Covid-19: Epidemiological Evidence

Since the first reports of covid-19 in Wuhan, China, high frequencies of diabetic individuals among hospitalized patients and those with fatal outcome have shown the importance of DM as a risk factor. Currently, type 2 DM is followed by other risky conditions such as advanced age, obesity, hypertension and CVD.

A Chinese study of 1,099 individuals with covid-19 showed that among 173 with severe disease 23.7% had hypertension, 16.2% DM, 5.8% coronary heart disease, and 2.3% cerebrovascular disease^9^ . Another study with 140 patients admitted to the hospital reported similar rates, 30% of hypertension and 12% of DM^10^ .

The most robust one conducted in China assessed a total of 44,672 cases of covid-19^11^ . The fatality rate was 2.3%, which differs according to age and severity of manifestations. Death occurred in 14.8% of patients older than 80 years (208 out of 1,408), 8.0% between 70 and 79 years (312 out of 3,918) and in 49.0% of severe cases (1,023 of 2,087). Among those who died and had chronic diseases, 10.5% had CVD, 7.3% DM, 6.3% chronic respiratory disease, 6.0% hypertension, and 5.6% cancer. An analysis of 52 critical ill patients reinforced the role of age, male sex (67%) and previous chronic disease (40%) as risk factors^12^ . Authors reported that non-survivors at 28 days (n = 32) neither presented difference in relation to the time from the onset of symptomatology, type of symptoms nor radiological changes until hospitalization. Non-survivors and survivors differed by age (64.6 versus 51.9 years, respectively) and proportion of concomitant chronic diseases (53% versus 20%).

Factors associated with mortality were also analyzed in 191 severe cases in China; 54 died and 137 discharged^13^ . The strongest association was found with coronary heart disease (OR 21.4, 95%CI 4.6-98.8), but also hypertension (OR 3.0, 95%CI 1.6-5.9), DM (OR 2.9, 95%CI % 1.3-6.1), COPD (OR 5.4, 95%CI 1.0-30.4), and multiple organ failure (OR 6.1, 95%CI 3.5-10.8) which increase the risk of death. Altered circulating biomarkers (ferritin, interleukin 6, D-dimer, troponin) were associated with fatal outcome. Considering the total of risk markers, independent predictors of mortality were older age, CVD, multiple organ failure and D-dimer concentration >1 mcg/mL^13^ . Notably, DM and hypertension could have not appeared as independent predictors considering that they are mediators of cardiovascular events and mortality, which should imply underestimation of the effect of these diseases for the fatal outcomes^13^ .

As the pandemic advanced to other continents, data from Europe and Americas have confirmed this worrying relationship between DM and covid-19 prognosis. In the Lombardy region of Italy, among 1,591 ICU patients affected between February and March 2020, 68% had at least one comorbidity, 49% systemic hypertension, 21% CVD, 17% type 2 DM, 8% cancer, and 4% COPD^14^ .

In the USA, the Coronavirus Disease 2019–Associated Hospitalization Surveillance Network (COVID-NET) showed that 89.3% of 178 adults had one or more underlying morbidities; the most common was hypertension (49.7%), followed by obesity (48.3%), chronic lung disease (34.6%), DM (28.3%) and CVD (27.8%). In individuals aged between 18 and 49 years, obesity was the most frequent condition, followed by chronic lung disease (primarily asthma) and DM while in those aged between 50 and 64 years the most frequent diseases were hypertension and DM^15^ . Further report confirmed that age > 60 years with a BMI 30-34 kg/m^2^ doubled the chance of being admitted to hospital (HR 2.0, p < 0.0001) and critical care (HR 1.8; p = 0.006) compared to age < 60 years with a BMI < 30 kg/m^216^.

In Brazil, data from the Ministry of Health also identify DM and previous CVD as the comorbidities mostly associated with death in individuals affected by the SARS-Cov-2. DM and CVD can be seen as the same disease since atherosclerosis is present in the natural history of long-term DM. Also, hyperglycemia can firstly manifest during an acute cardiovascular event.

The concern about the relationship between cardiometabolic diseases and covid-19 is not unprecedented. Previous studies have improved knowledge on the associations of DM and CVD with influenza epidemics, H1N1 or MERS^17^ and this knowledge supports researchers to provide clues to manage the current pandemic.

The understanding of the biological plausibility for the association of DM and covid-19 might clarify underlying mechanisms, reinforcing possible causal relationships, thus enabling more effective prevention strategies to face viral epidemics.

### Advances in Plausibility of the Association between DM and Covid-19 Severity

Investigations on the biological plausibility of the association between DM and the severity of covid-19 are ongoing but it remains unclear.

Adequate glycemic control reduces predisposition and improves prognosis against infections in people with DM. A recent study, including individuals without DM, showed that high levels of fasting plasma glucose were a predictor of poor outcomes and death in covid-19 patients^18^ . Direct role of the disturbance of glucose metabolism and its contribution to the covid-19 severity has to be shown.

DM is a low-grade inflammation state, and a high-grade of systemic inflammation occurs in covid-19, reflected by elevations in inflammatory markers such as C-reactive protein, dimer-D and ferritin^13^ . Therefore, DM and its associated diseases could provide a background to exacerbate inflammatory process contributing to the progression of covid-19 in diabetic individuals.

In the same context, the association of obesity with covid-19 could corroborate this hypothesis considering that the former is also a pro-inflammatory state and a risk factor for type 2 DM. Excessive adiposity produce cytokines and generates insulin resistance and endothelial dysfunction, an early event in atherogenesis. Reasons why obesity has been associated with covid-19 hospitalization and severity^15^ , especially in people under 60 years of age^16^ are under discussion. As previously mentioned, atherosclerosis is the macrovascular complication of DM and its presence could contribute to understanding the increased covid-19 severity and mortality. In some studies, and also in Brazil (unpublished data), CVD has been considered the strongest predictor of severity and mortality due to SARS-Cov-2 infection. The effect of cardiovascular causes of death on hospitalized patients, including myocarditis, arrhythmias, myocardial infarction and systemic thrombosis is highlighted in recent review^17^ .

A possible deleterious role of medications to treat these morbidities was raised. Pathophysiological pathways of DM and hypertension were demonstrated to worsen SARS-Cov-2 infection^19^ and some medications mechanism of action were based on these pathways. Coronaviruses were able to bind to target cells through angiotensin-converting enzyme 2 (ACE2) receptor which is expressed in several tissues. Researchers documented that ECA2 is increased in individuals with type 1 and type 2 DM and that some medications can increase ACE2 expression, such as ACE inhibitors, angiotensin 2 receptor blockers (ARB), thiazolidinediones and ibuprofen. However, no evidence indicates potential adverse effects of these agents on the prognosis of diabetic and hypertensive individuals with covid-19. Therefore, scientific societies such as the Brazilian Diabetes Society^20^ have not recommended the withdrawal of these drugs due to SARS-Cov-2 infection^21 , 22^ .

## FINAL CONSIDERATIONS

While advances in the knowledge of the relationship between DM and covid-19 occur, health professionals have experienced difficulties in clinical management and public health systems in the support of the heavy burden of these two morbidities in populations. Pathophysiological relationship must be well understood to plan preventive measures and/or to propose more effective therapeutic strategies. In this context, we could indicate some gaps regarding the severity of DM and SARS-Cov-2 infection that should be focused in future studies. It is possible that susceptibility and prognosis vary according to the type of DM, age, duration of illness, degree of glycemic control, presence of complications and medications in use.

In summary, DM in individuals with covid-19 is more than the simple sum of two morbidities in prognostic terms. Understanding underlying mechanisms of this association are urgently necessary to face the current SARS-Cov-2, to propose in effective prevention through vaccination and pharmacological agents as well as to prepare the health care systems for future challenges.
